# The Association Between the Developing Nasal Microbiota of Hospitalized Neonates and *Staphylococcus aureus* Colonization

**DOI:** 10.1093/ofid/ofz062

**Published:** 2019-03-21

**Authors:** Dina F Khamash, Emmanuel F Mongodin, James R White, Annie Voskertchian, Lauren Hittle, Elizabeth Colantuoni, Aaron M Milstone

**Affiliations:** 1 Division of Pediatric Infectious Diseases, Department of Pediatrics, Johns Hopkins University School of Medicine, Baltimore, Maryland; 2 Institute for Genome Sciences, University of Maryland School of Medicine, Baltimore, Maryland; 3 Resphera Biosciences, Baltimore, Maryland; 4 Department of Biostatistics, Bloomberg School of Public Health, Johns Hopkins University, Baltimore, Maryland

**Keywords:** microbiome, nasal cavity/microbiology, sequence analysis, staphylococcal infections, *Staphylococcus aureus*

## Abstract

**Background:**

Hospitalized neonates are at high risk for invasive *Staphylococcus aureus* infections. *S. aureus* nasal colonization often precedes infection. The nasal microbiota may preclude or support colonization. We aimed to characterize and compare the nasal microbiota of hospitalized neonates who acquire *S. aureus* colonization (cases) and those who do not acquire *S. aureus* (controls).

**Methods:**

We obtained residual nares samples from hospitalized neonates who were screened weekly for *S. aureus* nasal colonization and treated with intranasal mupirocin if colonized. Eight cases were matched based on chronologic age and systemic antibiotic exposure to 7 controls. We extracted DNA, sequenced the V3-V4 region of the 16s rRNA gene, and performed taxonomic assignments. The bacterial species richness, relative abundance, and in silico predicted gene content were compared between cases and controls at 7 days before *S. aureus* acquisition, at the time of acquisition, and 7 days after acquisition and treatment.

**Results:**

Common commensals including nondiphtheriae corynebacteria were more abundant in the nares of controls and *Rothia mucilaginosa* was more abundant in cases 7 days after intranasal mupirocin treatment than in cases 7 days before *S. aureus* acquisition. Controls and treated cases had a higher predicted abundance of genes contributing to the synthesis of certain antimicrobial compounds than in cases before *S. aureus* acquisition.

**Conclusions:**

Neonates without *S. aureus* nasal colonization had a higher abundance of bacterial species that antagonize *S. aureus* directly or by selecting for beneficial co-colonizers. These differences may inform novel *S. aureus* infection prevention strategies in high-risk infants.

*Staphylococcus aureus* is a leading cause of invasive bacterial infections in hospitalized infants [1]. Asymptomatic colonization of the anterior nares, the primary anatomic niche for *S. aureus*, is an important precursor for *S. aureus* disease [2]. Many neonates become colonized by *S. aureus* shortly after birth.

Bacterial species inhabiting the nasal cavity may affect an infant’s susceptibility to acquiring *S. aureus* colonization and subsequent risk of *S. aureus* infection [3]. The growing accessibility of sequencing technologies has made it possible to explore these microbial communities and how they interact to promote susceptibility or resistance to *S. aureus* colonization. In adults, some organisms are found more commonly in noncarriers of *S. aureus*, and introduction of these organisms may eliminate *S. aureus* from the nasal microbial community or limit its growth [4].

Not all neonates exposed to *S. aureus* become colonized. The complex association between the nasal microbiome and *S. aureus* colonization in high-risk neonates has not been previously explored. Using 16S rRNA gene sequencing, we aimed to characterize the developing nasal microbiota in a convenience sample of hospitalized infants over time, compare the microbial communities inhabiting the nares of those neonates who did and did not acquire *S. aureus* colonization, and infer the functional pathways present in these microbial communities that may impact *S. aureus* colonization.

## METHODS

### Study Design and Population

Our study describes the neonatal nasal microbiota and its association with *S. aureus* colonization in a convenience sample of 15 neonates admitted to the Johns Hopkins Hospital neonatal intensive care unit (NICU). This study was approved by the Johns Hopkins Medicine Institutional Review Board. All infants admitted to the NICU have weekly swabs collected from the anterior nares to detect *S. aureus* colonization using Eswabs (Copan diagnostics, Murrieta, CA), as previously described [5]. Neonates who grow *S. aureus* on weekly surveillance cultures are treated with intranasal mupirocin for 5 days. We considered cases to be infants who acquired *S. aureus* colonization, defined as having at least 1 surveillance culture grow *S. aureus* during a NICU admission. Time 0 (time = 0) was set as the collection date at which the first surveillance culture grew *S. aureus*. Control neonates were selected if they had a swab collected at a chronological age within ±7 days of the age of a case at time = 0 and had no positive surveillance cultures for *S. aureus.* As all neonates included in this study were admitted to the NICU at birth, we considered chronological age equivalent to length of NICU stay. In addition to chronologic age at the time of sample collection, cases and controls were matched based on their exposure to systemic antibiotics before time = 0 (Table 1). One control was matched to 2 cases, and the time = 0 control samples matched to each case differed based on chronologic age at the time of *S. aureus* acquisition (Table 1). Therefore, 15 neonates contributed 8 age-matched pairs at time = –7 and time = 0 and 6 age-matched pairs at time = +7 for analysis.

**Table 1.** **Study Cohort Characteristics**

Matched Pair	Patient	Acquired *S. aureus*	Delivery Mode	Sex	Gestational Age, wk	Birth Weight, g	Chronologic Age at Time = 0, d	Systemic Antibiotic Exposure
1	A	Yes	Cesarean	M	28–32	<1500	35	Exposure >14 d before t = 0
	B	No	Vaginal	F	<37	<2500	28	
2	C	Yes	Cesarean	F	<28	<1000	33	Exposure within 14 d of t = 0
	D	No	Cesarean	F	<28	<1000	33	
3	E	Yes	Cesarean	M	28–32	<2500	15	No exposure
	F^a^	No	Cesarean	M	<37	<2500	16	
4	G	Yes	Cesarean	M	<37	<2500	59	Exposure >14 d before t = 0
	H	No	Cesarean	F	<28	<1000	56	
5	I	Yes	Cesarean	F	28–32	<1500	22	Exposure >14 d before t = 0
	J	No	Cesarean	F	<37	<2500	24	
6	K	Yes	Cesarean	F	28–32	<1000	29	No exposure
	F^a^	No	Cesarean	M	<37	<2500	23	
7	L	Yes	Vaginal	M	28–32	<1000	17	Exposure within 14 d of t = 0
	M	No	Vaginal	F	<37	<2500	17	
8	N	Yes	Cesarean	M	<28	<1000	38	Exposure >14 d before t = 0
	O	No	Cesarean	M	<37	<2500	42	

^a^Patient F was matched to 2 cases: patient E and patient K.

### Sample Processing and DNA Extraction

As part of an ongoing hospital surveillance program, surveillance specimens were plated on CHROM agar plates (BD Diagnostics, Sparks, MD) and incubated for 24 hours, after which *S. aureus* was identified by Gram stain and coagulase test, as previously described [6]. Residual Amies transport media was transferred to cryovials and stored at –80°C. For microbial community DNA extraction, select samples were thawed, and cells were lysed using an enzymatic cocktail of lysozyme, mutanolysin, proteinase K, and lysostaphin followed by mechanical lysis using the bead-beating method. The extracted DNA was then purified using the Zymo Fecal DNA kit (Zymo Research, Irvine, CA) to provide high-quality genomic DNA for bacterial community profiling. Polymerase chain reaction (PCR) amplification of the V3-V4 hypervariable region on the 16S rRNA gene and sample multiplexing for high-throughput sequencing with the Illumina HiSeq 2500 (300-bp paired-end reads) using the universal primers 319F and 806R and a dual-indexing strategy was performed as previously described [7]. Gel electrophoresis was used to confirm the presence of amplicons, after which normalization was performed (25 ng of 16S rRNA gene amplicon pooled for each sample) using the SequalPrep Normalization Plate Kit (Thermo Fisher Scientific, Waltham, MA). Negative controls were included at each processing step (extraction, 16S rRNA gene PCR amplification, and sequencing). A total of 89 samples were characterized in this study. The sequences generated and analyzed in this study were deposited in GenBank, linked to NCBI BioProject ID PRJNA476107.

### 16S rRNA Amplicon Sequence Analysis

Raw paired reads were merged using FLASH and filtered for a maximum error rate of 0.5% and a minimum length of 200 bp using Trimmomatic and QIIME [8–10]. BLASTN was then used to identify and remove spurious hits to the PhiX control genome. Primers were trimmed from the resulting sequences, which were then evaluated for chimeras using UCLUST in de novo mode [11]. Sequences were then screened for human contaminants using Bowtie2, followed by a sensitive BLASTN search against the GreenGenes 16S database [12]. Chloroplast and mitochondrial contaminants were identified and filtered with the RDP classifier with a confidence threshold of 80% [13]. Resulting high-quality sequences were then submitted for high-resolution taxonomic assignment with Resphera Insight, an approach capable of providing species-level assignments [14]. Sequences were also analyzed using Phylogenetic Investigation of Communities by Reconstruction of Unobserved States (PICRUSt) with default parameters to infer functional gene content of each sample [15].

### Statistical Analysis

We compared the bacterial species richness, composition, and in silico predicted gene content of the nasal microbiota at weekly intervals for neonates who did and did not acquire *S. aureus* colonization. After normalization of coverage levels to 30 000 sequences per sample, the number of observed species was used as a measure of bacterial richness and was compared between cases and controls using the Mann-Whitney test separately for samples taken the week before *S. aureus* acquisition (time = –7 days), at the time of *S. aureus* acquisition (time = 0), and postcolonization and treatment with intranasal mupirocin (time = 7 days). For each assessment time (time = –7, 0, and 7), the relative abundance of select organisms was compared between cases and controls assuming a negative binomial distribution with *P* value adjustment using the false discovery rate (FDR). The mean change in relative abundance of bacterial species in cases pre-acquisition (time = –7) and post-treatment (time = +7) was computed, and the distribution of relative abundance in cases at these 2 time points was compared using the Wilcoxon signed rank test. Permutational analysis of variance using the R package vegan was used to evaluate the impact of interindividual variation, relative time, and case–control status on microbial community differences across 3 β-diversity measures: Weighted UniFrac, Unweighted UniFrac, and Bray-Curtis dissimilarity (Supplementary Table 1) [16]. We report the *R*^2^ and *P* values for Bray-Curtis dissimilarity below.

## RESULTS

### Study Population

The study cohort was comprised of 15 neonates who underwent weekly testing for nasal *S. aureus* colonization, 8 of whom acquired *S. aureus* during the study period (Table 1). Of these 15 neonates, 8 (53%) were female, 12 (80%) were born by cesarean section, and 12 (80%) were exposed to systemic antibiotics before time = 0. All of the infants included in our study were born preterm (<37 weeks gestational age) with a median gestational age of 30 weeks and had low birth weights (<2500 g), with the median birth weight being 1380 g. We performed 16s rRNA gene sequencing on microbial communities from 89 nasal swabs, yielding 6.2 × 10^6^ reads.

### The Microbiota of Hospitalized Neonates Exhibits Temporal Variability

**Figure 1.**
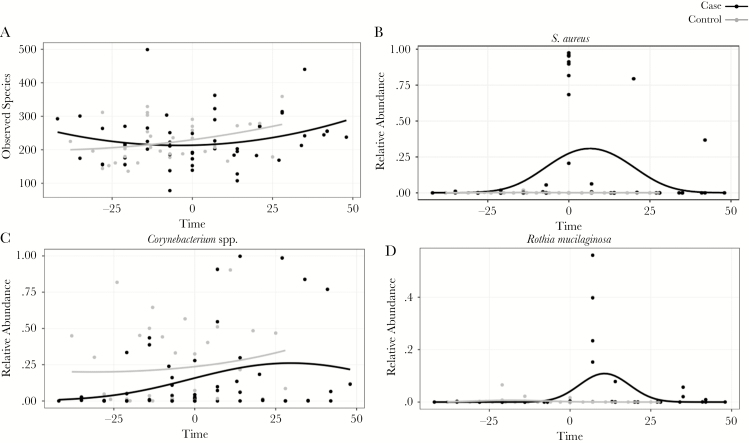
Variability of the neonatal nasal microbiota in neonates who acquired *Staphylococcus aureus* nasal colonization (cases) and those who did not acquire *S. aureus* nasal colonization (controls) over time (days) relative to the day of acquisition for cases (t = 0). A, Species richness. B, Relative abundance of *S. aureus.* C, Relative abundance of *Corynebacterium* species. D, Relative abundance of *Rothia mucilaginosa*.

The number of observed species or species richness was compared between cases and controls over time (Figure 1A). Temporal changes in the proportion of organisms that were differentially abundant between cases and controls were also analyzed. The changes in the relative abundance of select organisms of interest: *S. aureus*, *Corynebacterium* species, and *Rothia mucilaginosa* are shown over time in Figure 1B–D. Sample time point was a significant contributor to compositional variability in the nasal microbiota (*P* < .01; *R*^2^ = .03). Cases had a significantly lower richness at time = 0 than neonates of the same chronological age who were not colonized by *S. aureus* (*P* = .02), but the 2 groups were comparably rich at all other measured time points (Figure 1A). At time = 0, *S. aureus* was the most abundant species in all cases; however, all 15 neonates studied had a low but detectable abundance of *S. aureus* before time = 0 (Figure 1B). Controls were more enriched with *Corynebacterium* species than cases for most time points analyzed (Figure 1C).

Cases treated with intranasal mupirocin (time = +7) were comparably rich as controls at time = +7 (*P* = .75). The mean relative abundance of *Corynebacterium* species in cases post-treatment at time = +7 increased to 29.4% from a mean of 9.2% at time = –7 (mean difference, 20.2%; 95% confidence interval [CI], 10.64% to 51.02%; *P* = .07 by Wilcoxon signed rank sum). The mean relative abundance of *Corynebacterium* species in cases post-treatment became similar to that of controls at time = +7 (29.4% and 24.5%, respectively; mean difference, 4.9%; 95% CI, –32.7% to 42.2%; *P* = 1.0 by Wilcoxon rank sum). The mean relative abundance of *Rothia mucilaginosa* in cases post-treatment, at time = +7, increased to 22.5% from a mean of <0.1% in cases before acquisition of *S. aureus* colonization (mean difference, 22.5%; 95% CI, –0.89% to 45.81%; *P* = .03 by Wilcoxon signed rank sum). At time = +7, cases also had a higher mean relative abundance of *Rothia mucilaginosa* than controls (22.5% and 0.4%, respectively; mean difference, 22.1%; 95% CI, 1.8% to 42.3%; *P* = .02 by Wilcoxon rank sum).

### Cases and Controls Have Compositionally and Functionally Distinct Nasal Microbiotas

**Figure 2.**
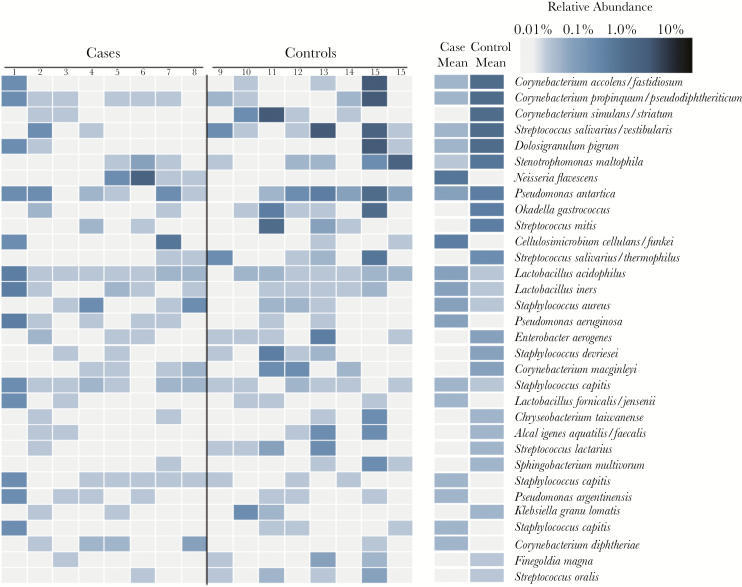
Relative abundance of species in specimens taken from neonates who acquired *Staphylococcus aureus* nasal colonization (cases) and those who did not acquire *S. aureus* nasal colonization (controls) in the week before cases acquired *S. aureus* (time = –7 days). Also shown are the mean relative abundance of species in cases and controls at time = –7.

Interindividual variation was the dominant factor contributing to the composition of the nasal microbiota (*P* < .01; *R*^2^ = .30), but case–control status alone also contributed significantly to the variation in composition observed between neonates (*P* < .01; *R*^2^ = .04). The mean relative abundance for each observed species was compared between cases and controls for the samples taken 1 week before case colonization (time = –7) (Figure 2). Overall, 136 species were differentially abundant (FDR-adjusted *P* < .05) between cases and controls. Controls had a significantly higher mean relative abundance of several *Corynebacterium* species, including *C. propinquum/pseudodiphtheriticum*, *C. accolens/fastidiosum*, and *C. simulans/striatum*. Viridans streptococci, including *Streptococcus salivarius/vestibularis*, *Streptococcus lactarius*, and *Streptococcus oralis,* were significantly more abundant in controls than cases as well. Controls also had a higher mean relative abundance of *Dolosigranulum pigrum*, a Gram-positive coccus that often inhabits the respiratory tract. Cases had a higher average abundance of pathobionts like *Pseudomonas aeruginosa*, *Haemophilus influenzae*, and *Corynebacterium diphtheria*e in the week before acquisition of *S. aureus* colonization.

**Figure 3.**
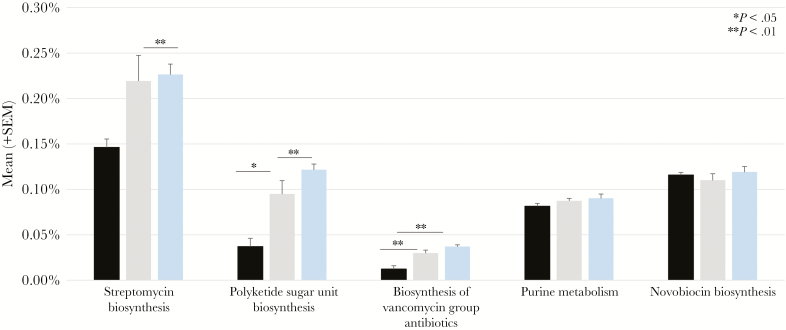
Predicted mean relative abundance of genes contributing to select functional pathways in neonates who acquired *Staphylococcus aureus* nasal colonization (cases) before *S. aureus* acquisition at time = –7, matched neonates who did not acquire *S. aureus* nasal colonization (controls) at time = –7, and cases post-treatment at time = +7.

PICRUSt was used to infer functional gene content in cases and controls using samples taken at time = –7 (Figure 3). Only select pathways related to the synthesis of antimicrobial substances including vancomycin group antibiotics are discussed, as the association of other functional pathways to bacterial fitness remains unclear. In controls, the mean proportion of predicted genes contributing to the synthesis of certain antimicrobial compounds, including streptomycin, vancomycin group antibiotics, and polyketide sugars, many of which have antimicrobial activity, was higher than in cases. The relative abundance of genes contributing to the synthesis of these antimicrobial compounds in cases after treatment with mupirocin (time = +7) was significantly higher than in cases before treatment (time = –7) and was similar to controls (time = +7).

## DISCUSSION

We studied the nasal microbiota of hospitalized neonates and identified significant differences between the composition and predicted metagenome of neonates who did and did not acquire *S. aureus*. Neonates are exposed to a unique environment and interventions such as antibiotics and respiratory support in the NICU. These exposures, along with the tolerance of the neonatal immune system, result in high intra-individual and temporal variability in resident bacteria [17, 18]. Although we show that the neonatal microbiota changed over time, interindividual variation played a dominant role in dictating microbial community composition, which may indicate that infants develop a distinct nasal microbiota in the first weeks of life. Our findings suggest that antagonistic relationships between *S. aureus* and potentially protective commensals may contribute to *S. aureus* colonization resistance in certain hospitalized neonates despite environmental exposures and time-dependent variability.

Infants who did not acquire *S. aureus* colonization had a higher abundance of organisms commonly present in *S. aureus* noncarriers, such as *D. pigrum*, when compared with cases [19]. The relative abundance of *D. pigrum* is negatively associated with the likelihood of *S. aureus* colonization in adults, suggesting an antagonistic relationship between the 2 organisms. *D. pigrum* alone does not outcompete *S. aureus* but often co-colonizes the nasal cavity with certain *Corynebacterium* species. We found that this relationship may also exist in hospitalized neonates, as controls were also more enriched with *Corynebacterium* species, including *C. propinquum/pseudodiphtheriticum*, *C. simulans/striatum,* and *C. accolens/fastidiosum.* Nondiphtheriae *Corynebacterium* species have been shown to eliminate *S. aureus* nasal colonization in adult studies, though the mechanisms behind these interactions remain unclear [20]. Viridans group streptococci, which demonstrated the ability to eliminate *S. aureus* through hydrogen peroxide production, were also more enriched in controls than cases [21].

Although all 15 patients included in this study had a low abundance of *S. aureus* before time = 0, only 8 neonates proceeded to have an abundance corresponding to detection by conventional culture, a known risk factor for *S. aureus* infection. Detection of *S. aureus* in samples that had negative cultures may represent taxonomic misclassification or a low absolute abundance of *S. aureus* that is undetectable by culture. Neonates who had cultures growing *S. aureus* had a lower species richness at the time of colonization than controls, and this also corresponded to the point at which *S. aureus* was the most abundant organism in the nares for these patients. This may suggest that lower richness predisposed the nares of certain neonates to the domination of *S. aureus* or that *S. aureus* dominance precludes the survival of more diverse communities. *Corynebacterium* species have been shown to alter gene expression to limit *S. aureus* growth, decrease fitness, and attenuate virulence, suggesting that a higher relative abundance of nondiphtheriae corynebacteria may have limited the abundance of *S. aureus* in controls [4].

After neonates with cultures growing *S. aureus* were treated with intranasal mupirocin, they became more species rich overall, with a higher abundance of *Corynebacterium* and *Rothia* species. The predicted abundance of genes contributing to the synthesis of certain antimicrobial compounds more abundant in controls also increased in cases post-treatment, reflecting these changes in composition. Although the role of these antimicrobial compounds in *S. aureus* nasal colonization remains unclear, the roles of antimicrobial peptides in direct bacterial interference against *S. aureus* in other anatomic sites have recently been described. *Bacillus subtilis* and *Staphylococcus caprae* produce peptides that interfere with the agr-mediated quorum sensing needed for *S. aureus* colonization and have been shown to inhibit intestinal and dermal *S. aureus* colonization, respectively [22, 23]. A similar mechanism serves as 1 of many bacterial interactions that limit the growth of *S. aureus* in the nares.

This study has several limitations. First, a small convenience sample of neonates was studied, each having a different number of samples collected at different times throughout admission. Second, case–control matching did not account for characteristics such as birth mode, respiratory support, and diet. These characteristics may impact an infant’s susceptibility to *S. aureus* acquisition directly or by altering the nasal microbiome.

Despite these limitations, we found significant differences between the neonatal microbiota of cases and controls preceding *S. aureus* colonization that were largely consistent with previous studies. Controls had a higher abundance of bacterial taxa that have been shown to antagonize *S. aureus* either directly or by selecting for co-colonizers that support a temporally stable and diverse microbiota. These results support speculations about the role of candidate commensals in promoting colonization resistance to *S. aureus*. Further investigation is warranted to better establish the identity and role of protective species and their bacterial peptides in the developing nasal microbiota of hospitalized infants at high risk for invasive *S. aureus* infections.

## Supplementary Data

Supplementary materials are available at *Open Forum Infectious Diseases* online. Consisting of data provided by the authors to benefit the reader, the posted materials are not copyedited and are the sole responsibility of the authors, so questions or comments should be addressed to the corresponding author.

Supplementary_Table_1

## Notes

***Prior presentation. ***Preliminary results for this study were presented at IDWeek; October 2017; San Diego, CA.
