# Supplementary material for: The Association Between the Developing Nasal Microbiota of Hospitalized Neonates and Staphylococcus aureus Colonization
Source: Open Forum Infect Dis. 2019 Mar 21;6(4):ofz062. doi: 10.1093/ofid/ofz062 (PMC6441571; doi:10.1093/ofid/ofz062)
Supplement: Supplementary_Table_1 [file ofz062_suppl_supplementary_table_1.docx]

Supplementary Table 1.

| **Beta-Diversity Distance Measure*** | **Factor^** | **R^2^** | ***p*-value** |
| --- | --- | --- | --- |
| Bray-Curtis | Time-point | 0.03464 | 0.001 |
| Bray-Curtis | Case-Control Status | 0.03986 | 0.001 |
| Bray-Curtis | Patient ID | 0.29533 | 0.001 |
|  |  |  |  |
| Unweighted UniFrac | Time-point | 0.01687 | 0.008 |
| Unweighted UniFrac | Case-Control Status | 0.01569 | 0.016 |
| Unweighted UniFrac | Patient ID | 0.18637 | 0.001 |
|  |  |  |  |
| Weighted UniFrac | Time-point | 0.01873 | 0.0848 |
| Weighted UniFrac | Case-Control Status | 0.02503 | 0.0529 |
| Weighted UniFrac | Patient ID | 0.35336 | 0.001 |
